# ID 128 - Custo-Efetividade da Terapia Preemptiva versus Terapia Empírica para Manejo de Infecções Fúngicas Invasivas em Pacientes com Neutropenia Febril no Sistema Único de Saúde

**DOI:** 10.5327/2237-9622.2025.v34s1.128

**Published:** 2025-11-25

**Authors:** Luiza Ikeda Seixas Cardoso, Daniel da Silva Pereira Curado, Juliana Machado-Rugolo, Ricardo de Souza Cavalcante

**Keywords:** custo-efetividade, terapia preemptiva, terapia empírica, neutropenia febril, infecção fúngica invasiva, aspergilose invasiva

## Abstract

**Introdução:**

As infecções fúngicas invasivas (IFI) são uma importante complicação em pacientes com neutropenia febril (NF). Com o uso de antifúngicos profiláticos, a aspergilose invasiva tornou-se uma das principais IFI neste cenário clínico. Há duas formas de manejo destes pacientes. Uma delas é a terapia empírica, em que se introduz antifúngicos nos pacientes com NF com duração maior ou igual a quatro dias em uso de antibióticos de amplo espectro. Outra estratégia é a preemptiva, na qual se utiliza de biomarcadores (dosagem de galactomanana (GMN) e o teste molecular da reação em cadeia da polimerase (PCR) para um diagnóstico precoce e dirigido e uso mais restrito de antifúngicos. Um dos pontos centrais acerca da estratégia preemptiva são os custos que esta abordagem pode acarretar. Os testes de GMN e PCR apresentam custos diretos mais elevados que os exames rotineiros nos laboratórios e necessitam ser realizados de forma seriada. Esta condição leva a uma dúvida sobre a viabilidade econômica da incorporação destas tecnologias. Por outro lado, a terapia preemptiva permite um uso mais restrito de antifúngicos, o que leva a uma redução significativa de gastos e evita a resistência destes antimicrobianos. Portanto, o objetivo foi comparar a custo-efetividade do manejo empírico vs o preemptivo para infecções fúngicas invasivas em pacientes com neutropenia febril, na perspectiva do sistema público de saúde brasileiro.

**Método:**

Foi utilizado um modelo analítico de árvore de decisão, com um horizonte temporal de um ano, que teve como população-alvo os pacientes com idade acima de 18 anos, com neoplasias hematológicas e neutropenia febril. Para a estratégia preemptiva, foi considerado o monitoramento combinado com dosagem sérica GMN e pesquisa de Aspergillus spp por PCR. Os dados de desfechos e parâmetros clínicos para cada uma das estratégias avaliadas foram obtidos de estudos prévios publicados para a construção do modelo analítico. A medida de efetividade foi ano de vida salva. Os custos foram coletados em reais, obtidos da tabela de custos do SUS (Sigtap) e da média ponderada das compras realizadas nos últimos 18 meses pelas instituições públicas do Brasil e não foram aplicados ajustes pela inflação. Uma análise de sensibilidade determinística de uma via e uma probabilística, pela simulação de Monte Carlo de segunda ordem, foi realizada para avaliar as variações do modelo.

**Resultados:**

O modelo analítico estimou uma sobrevida de 91,2% nos pacientes manejados de forma preemptiva comparados a 89,5% na empírica. A estratégia preemptiva (R$ 14.908,80) gerou um custo final por paciente menor que a empírica (R$ 15.417,10). A razão de custo-efetividade incremental (RCEI) foi -R$ 28.238,89/ano de vida salva. A análise de sensibilidade determinística mostrou que todas as variações encontravam-se abaixo da disposição a pagar. Na análise probabilística, 91,23% das 10 mil simulações ficaram abaixo da disposição a pagar.

**Conclusão:**

Um importante achado deste estudo foi o menor custo final na estratégia preemptiva, que permitiu uma economia estimada de R$ 508,30 por paciente. Um resultado semelhante foi encontrado por outros autores, como Barnes e colaboradores, que avaliaram a custo-efetividade na perspectiva do sistema de saúde pública do Reino Unido, e encontraram uma economia de £ 740,64. Este estudo demonstra que a estratégia preemptiva foi mais custo-efetiva que a empírica, o que permite sua incorporação no sistema de saúde pública do Brasil.

